# Supplementary material for: Implications of GPIIB-IIIA Integrin and Liver X Receptor in Platelet-Induced Compression of Ovarian Cancer Multi-Cellular Spheroids
Source: Cancers (Basel). 2024 Oct 19;16(20):3533. doi: 10.3390/cancers16203533 (PMC11506604; doi:10.3390/cancers16203533)
Supplement: Supplementary file 1 [file cancers-16-03533-s001.zip › Supplementary Figures.pdf]

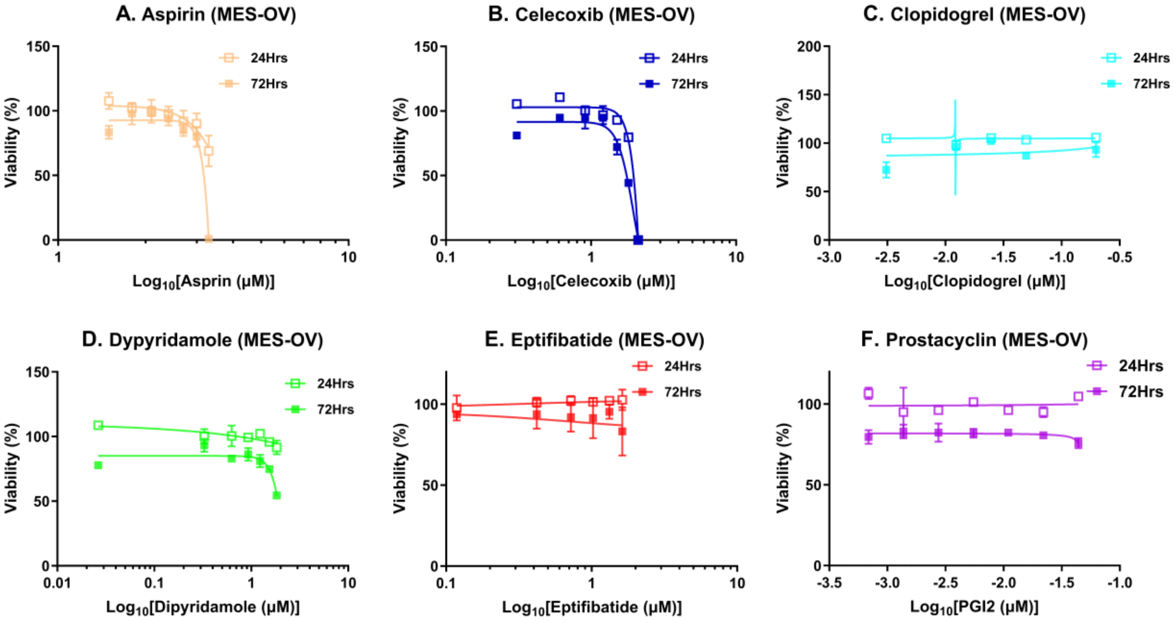

Figure S1. Platelets inhibitors cytotoxicity in MES-OV ovarian cancer cells: MTT assay was used to determine cell viability at 24 and 72 hours post treatment with platelet inhibitors at various concentrations.

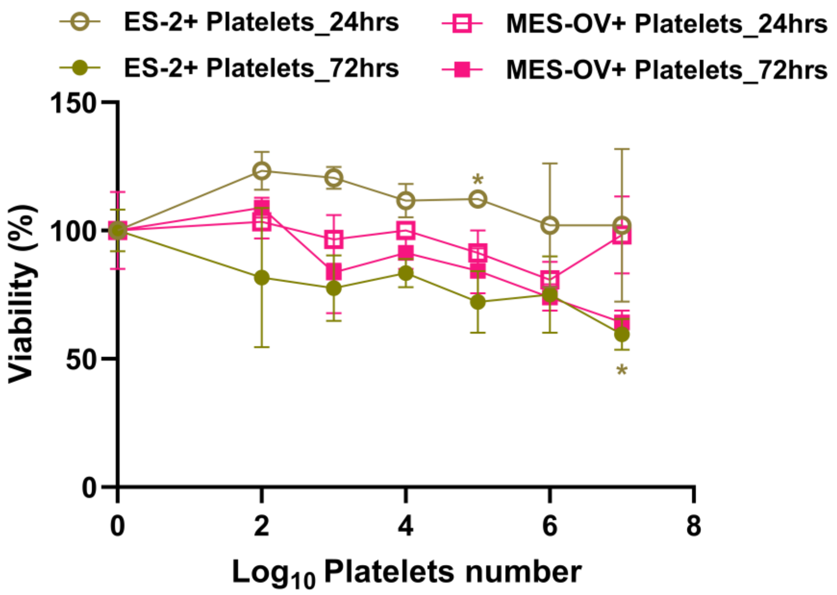

Figure S2. Effect on platelets on ovarian cancer monolayer cells: MTT assay was used to determine cell viability at 24 and 72 hours in ES-2 and MES-OV ovarian cancer cells that have been co-incubated with platelet at various concentrations.

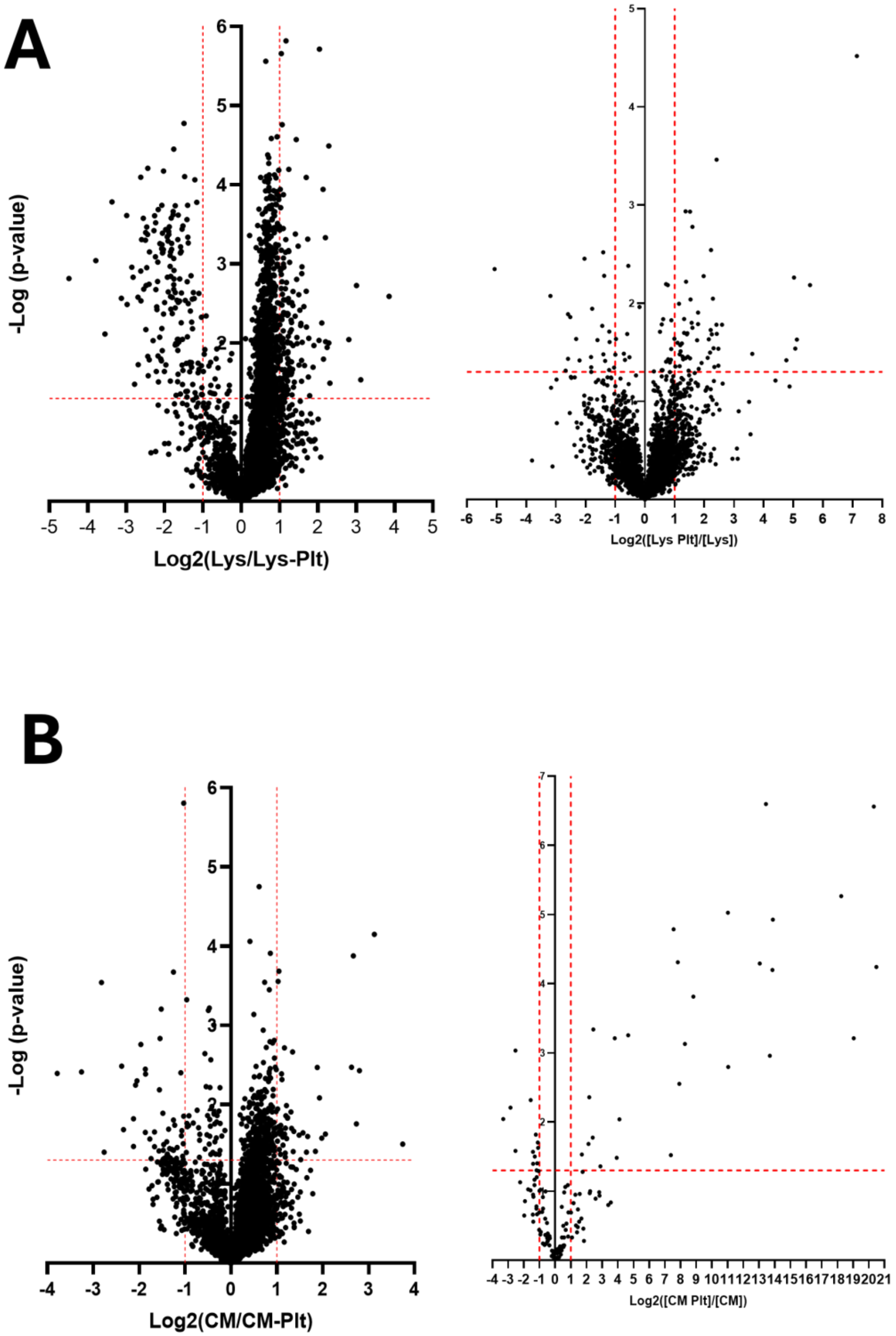

Figure S3: A. Volcano plots of molecules in spheroids-conditioned medium affected by platelets co-incubation with ovarian cancer spheroids. **B:** Volcano plots of molecules in spheroids lysates affected by platelets co-incubation with ovarian cancer spheroids. Molecules in spheroids lysates identified by mass spec analysis with the fold change of >1.5 and significant level of $p < 0.05$ were identified as being outside of the horizontal and vertical dashed red lines, respectively.
