# Supplementary material for: Implications of GPIIB-IIIA Integrin and Liver X Receptor in Platelet-Induced Compression of Ovarian Cancer Multi-Cellular Spheroids
Source: Cancers (Basel). 2024 Oct 19;16(20):3533. doi: 10.3390/cancers16203533 (PMC11506604; doi:10.3390/cancers16203533)
Supplement: Supplementary file 1 [file cancers-16-03533-s001.zip › Supplementary Table S1.pdf]

Table S1: Average spheroids diameter in μM: Magnetic ES-2 spheroids were co-incubated without platelets for control or with platelets and platelet inhibitors before imaging with Oxford Optronix GelCount to derive the diameter for each spheroid.

**Drug**	0hrs	1hr	16hrs
Control	1377	1461	1357
With Platelets	1372	837	783
Platelets + Aspirin	1359	1453	1373
Platelets + Celecoxib	1367	1302	1385
Platelets + Clopidogrel	1391	894	868
Platelets + Dipyridamole	1372	764	531
Platelets + Eptifibatide	1357	1431	1173
Platelets + Prostacyclin	1313	1180	1031
