# Supplementary material for: Implications of GPIIB-IIIA Integrin and Liver X Receptor in Platelet-Induced Compression of Ovarian Cancer Multi-Cellular Spheroids
Source: Cancers (Basel). 2024 Oct 19;16(20):3533. doi: 10.3390/cancers16203533 (PMC11506604; doi:10.3390/cancers16203533)
Supplement: Supplementary file 1 [file cancers-16-03533-s001.zip › Supplementary Table S2.pdf]

Table S2: Average spheroids density: Magnetic ES-2 spheroids were co-incubated without platelets for control or with platelets and platelet inhibitors before imaging with Oxford Optronix GelCount to derive the density for each spheroid.

**Drug**	0hrs	1hr	16hrs
Control	0.597	0.591	0.635
With Platelets	0.609	0.862	0.845
Platelets + Aspirin	0.616	0.573	0.629
Platelets + Celecoxib	0.601	0.800	0.596
Platelets + Clopidogrel	0.592	1.179	0.977
Platelets + Dipyridamole	0.599	1.087	1.267
Platelets + Eptifibatide	0.594	0.602	0.755
Platelets + Prostacyclin	0.590	0.473	0.552
